# Decellularized tissue-engineered heart valves calcification: what do animal and clinical studies tell us?

**DOI:** 10.1007/s10856-020-06462-x

**Published:** 2020-12-05

**Authors:** Adel F. Badria, Petros G. Koutsoukos, Dimosthenis Mavrilas

**Affiliations:** 1 Department of Fiber and Polymer Technology, Division of Coating Technology, KTH Royal Institute of Technology, Stockholm, Sweden; 2 Department of Mechanical Engineering and Aeronautics, Division of Applied Mechanics, Technology of Materials and Biomechanics, University of Patras, Patras, Greece; 3 Department of Chemical Engineering, University of Patras, Patras University Campus, 26504 Patras, Greece

## Abstract

Cardiovascular diseases are the first cause of death worldwide. Among different heart malfunctions, heart valve failure due to calcification is still a challenging problem. While drug-dependent treatment for the early stage calcification could slow down its progression, heart valve replacement is inevitable in the late stages. Currently, heart valve replacements involve mainly two types of substitutes: mechanical and biological heart valves. Despite their significant advantages in restoring the cardiac function, both types of valves suffered from serious drawbacks in the long term. On the one hand, the mechanical one showed non-physiological hemodynamics and the need for the chronic anticoagulation therapy. On the other hand, the biological one showed stenosis and/or regurgitation due to calcification. Nowadays, new promising heart valve substitutes have emerged, known as decellularized tissue-engineered heart valves (dTEHV). Decellularized tissues of different types have been widely tested in bioprosthetic and tissue-engineered valves because of their superior biomechanics, biocompatibility, and biomimetic material composition. Such advantages allow successful cell attachment, growth and function leading finally to a living regenerative valvular tissue in vivo. Yet, there are no comprehensive studies that are covering the performance of dTEHV scaffolds in terms of their efficiency for the calcification problem. In this review article, we sought to answer the question of whether decellularized heart valves calcify or not. Also, which factors make them calcify and which ones lower and/or prevent their calcification. In addition, the review discussed the possible mechanisms for dTEHV calcification in comparison to the calcification in the native and bioprosthetic heart valves. For this purpose, we did a retrospective study for all the published work of decellularized heart valves. Only animal and clinical studies were included in this review. Those animal and clinical studies were further subcategorized into 4 categories for each depending on the effect of decellularization on calcification. Due to the complex nature of calcification in heart valves, other in vitro and in silico studies were not included. Finally, we compared the different results and summed up all the solid findings of whether decellularized heart valves calcify or not. Based on our review, the selection of the proper heart valve tissue sources (no immunological provoking residues), decellularization technique (no damaged exposed residues of the decellularized tissues, no remnants of dead cells, no remnants of decellularizing agents) and implantation techniques (avoiding suturing during the surgical implantation) could provide a perfect anticalcification potential even without in vitro cell seeding or additional scaffold treatment.

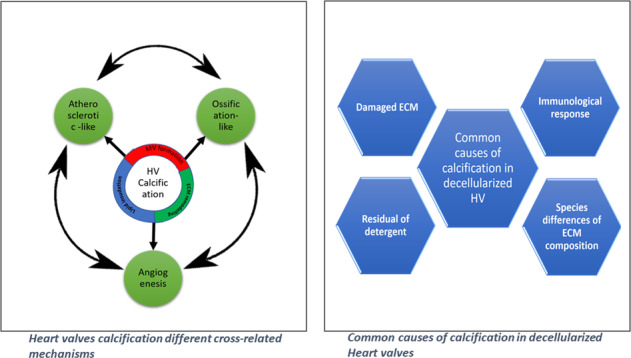

## Introduction

Cardiovascular diseases are the first cause of death worldwide. Among different heart malfunctions, like myocardial diseases, arterial atherosclerosis, aneurism and coronary artery infarction, native heart valve failure due to degenerative calcification is still a challenging problem. Calcification is divided into dystrophic and metastatic. While the first category is associated with localized damage or infection with no Ca/P abnormalities in the blood serum, the second one is more associated with an abnormal systematic level of Ca/P in serum due to certain diseases [1].

Heart valve calcification usually involves two stages: heart valves sclerosis (disease initiation) and valves stenosis (disease progression and valves obstruction) [2]. A long list of risk factors have been associated with this disease like genetic, age, smoking, hypertension, hyperlipidemia, etc. [3]. While drug-dependent treatment for the patients at risk or early stage of the calcification disease, like statins, may be sufficient, severe heart valve calcification makes their replacement inevitable in this late stage [4].

Nowadays there are two main types of heart valves substitutes: Mechanical and Bioprosthetic heart valves. Mechanical valves are mechanical devices from metals and/or polymers working as non-return flow valves. The main disadvantage of this type of valves is the formation of thrombotic clots in the stagnation points and/or hemolysis which requires lifelong anticoagulation therapy for the patients [5]. Bioprosthetic heart valves, on the other hand, consist of homograft or heterograft tissues taken from human or animals, usually pigs or cows, previously treated with various physicochemical processes for the amelioration of their performance. The main drawback related to these types of valve replacements is their propensity towards calcification upon prolonged contact with circulating blood [6].

To overcome the long-term malfunctions associated with both types of heart valve replacements, new types of heart valve substitutes have been developed over the past 20 years, generally known as tissue-engineered heart valves (TEHV) [7, 8]. In this type of substitute, biological tissues or synthetic biodegradable scaffolds are prepared and implanted to replace the diseased valve. In contrast to the permanent nondegradable substitutes, TEHV is a temporary degradable template that supports normal physiological valve function. In parallel, these templates assist in the gradual formation of a new living functional heart valve in synchronization with the gradual biodegradation of this scaffold material. Among different types of TEHV, the decellularized ones (dTEHV) have attracted the interest, because of their low cost, simple preparation protocols and their similarity to the native valves.

Despite the significant progress in this kind of dTEHV, they still can suffer from immunological rejection and calcification [9]. The latter problem (calcification) showed contradictory reports in the literature; while some reports demonstrated complete anticalcification effect of decellularization, others showed no significant effect (see section: effect of decellularization on calcification in animal and clinical studies).

In this review article we sought to find answers to the following questions: (1) Do decellularized heart valves calcify in vivo? (2) which factors can induce, reduce, or prevent the calcification process? (3) what are the possible mechanisms behind their calcification?

For this purpose, we did a retrospective study for all the English published literature of decellularized heart valves. Only animal and clinical studies were included in this review. Both animal and clinical studies were further subcategorized into four categories depending on the effect of decellularization on calcification: (A) Decellularization totally prevented calcification, (B) Decellularization reduced calcification, (C) Decellularization showed both pro-calcification and anticalcification potential based on the conditions involved and finally (D) Studies those do not mention clearly the effect of decellularization on calcification. All of those categories were tabulated and compared to each other’s depending on the host animal (in the case of animal studies), the type of heart valves involved, the technique of decellularization, recellularization and type of cells used, duration of the study follow-up, the size of the group involved and finally the techniques used to evaluate calcification occurrence. Due to the complex nature of calcification of heart valves, other in vitro and in silico studies were not included in this study. Finally, we compared the different results and summed up all the solid findings of whether decellularized heart valves calcify or not.

## Calcification of native heart valves

**Fig. 1:**
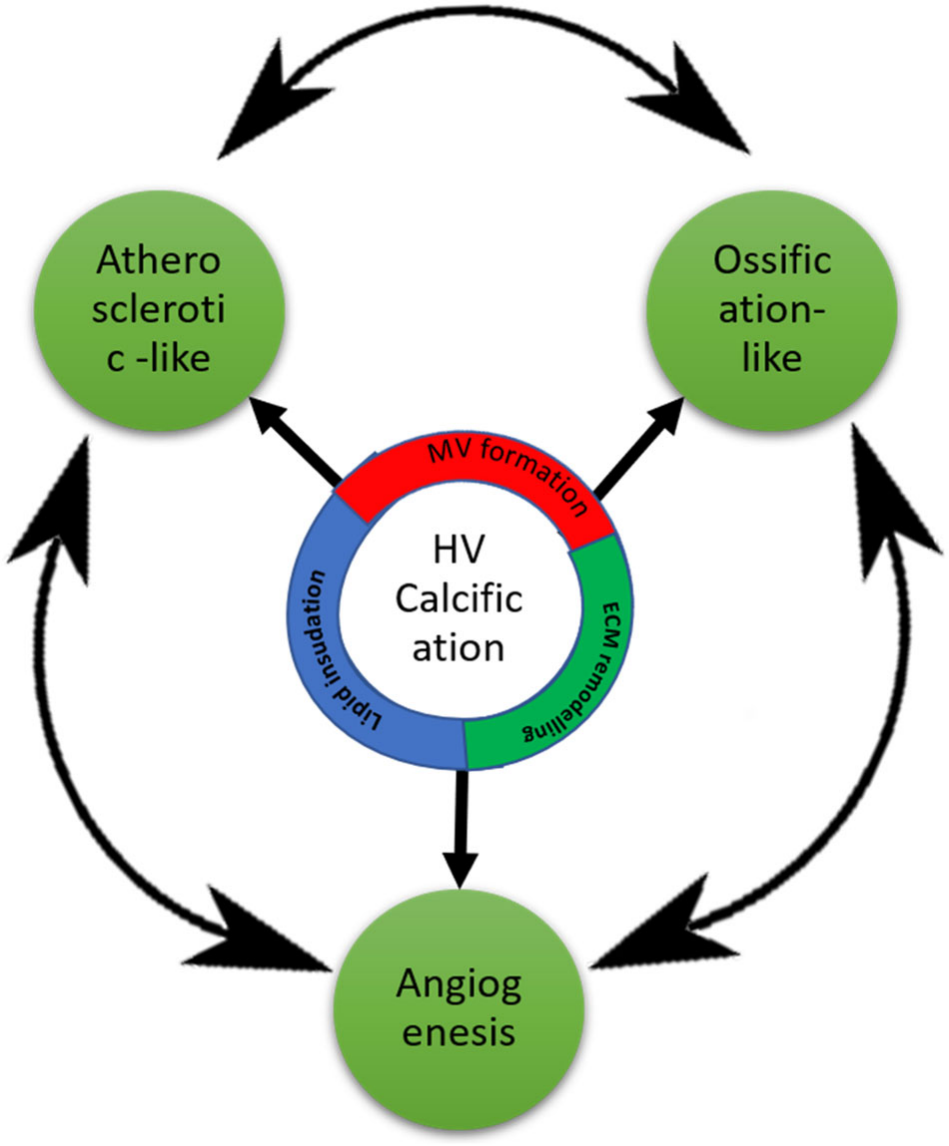
Heart valves calcification different cross-related mechanisms

In general, calcification of the native heart valves is a complex and poorly understood process. However, it appears to share a lot of similarities with different physiological and pathological conditions in the human body like ossification (cell differentiation and trans-differentiation) and atherosclerosis (immune-inflammatory responses) [10] as shown in Fig. 1.

## Cellular differentiation and trans-differentiation

In which the cellular activity results in calcification of the native heart valves. This can take place through either differentiation or trans-differentiation of surrounding cells. In the first one, mesenchymal progenitor cells differentiate into osteoblast-like cells and subsequently forms a bone-like matrix within the natural soft valvular tissue. While in the latter one, different kind of cells (like quiescent Vascular interstitial (VIC) and endothelial cells) transdifferentiate into osteoblast-like cells [11].

## Immune responses

In which the response of the immune system to different stimulations resulted in calcification. It takes place through two different cross-related mechanisms [10] as shown in Fig. 1:

(a) Atherosclerosis-like disease, according to which heart valve calcification can be considered an immune-inflammatory disease; consequently, all the atherosclerotic events will be seen in the heart valve calcification.

(b) Angiogenesis and neovascularization; although healthy human aortic valves are avascular structures, in calcified valves, there is evidence of angiogenesis near calcified nodules, under the leaflet border, and in areas infiltrated with inflammatory cells [10]. Calcified valves have been shown to contain small- and medium-sized microvessels as well as organized arterioles. Valve neovascularization is enhanced by the release of proangiogenic factors by inflammatory cells and/or by downregulation of inhibitors of angiogenesis [12]. Mast cells, which contain vascular endothelial growth factor were found to be present and degranulated in calcified valves [13]. Furthermore, in vitro studies confirmed that mast cells secrete factors that stimulate vascular endothelial growth factor production by myofibroblasts to augment local levels of this proangiogenic factor [10, 13].

Those two mechanisms exhibit their calcific action through one or more of the following ways:

1. Matrix Vesicle formation and microcalcific nodules
  In this calcification process, heart valve calcification is associated with matrix vesicles (MV) formation that serve as a nucleus for calcium deposition. Under normal physiological conditions, MV takes place in the process of the natural formation of bone and cartilage. On the other hand, under certain circumstances -like hypercalcemia- smooth muscle cells have shown a release of MV which could lead to microcalcific nodules formation. Noteworthy, such a process takes place under inflammation conditions suggesting that inflammation may be a considerable factor in the calcification process [14].
  This microcalcific nodule formation has also been shown to be a passive process meaning that no active cellular mechanisms are involved. This is supported by the presence of amorphous and crystalline calcific deposits at the sites of cell death, either by apoptosis or necrosis. Therefore, the residuals of the dead cells’ debris may be involved in the formation and propagation of calcific deposits. However, the underlying mechanism is not yet well understood [10]. Conclusively, the valve calcification through either passive or cellular mechanisms is very likely to be cross related. Also, it is possible that these osteoblast-like cells may actively generate vesicles or undergo apoptosis resulting in the accumulation of calcium and the subsequent development of microcalcific nodules [15].
2. ECM remodeling-mediated calcification
  A mentioned earlier, one of the mechanisms involved in heart valve calcification is the abnormal remodeling of valvular tissue ECM. This is often a consequence of increased expression of some metalloproteinases and cathepsins, which may result in the degradation of collagen and elastin and the formation of pro-inflammatory peptides, provoking the inflammatory response and allowing the expansion of calcific deposits. Additional evidence for this cellular mechanism is the observed activation of VIC in the calcified heart valves. It leads to abnormal secretion of collagen, hyaluronan, and other extracellular components, consequently affecting the stiffness of the valves and modulating the transition of VIC into osteoblast-like cells [10].
3. Lipid insudation

Atherosclerotic-like mechanism of calcification involves lipid accumulation on the formed calcific deposits, because of hypercholesterolemia. A significant relation has been found between the high levels of proatherogenic oxidized low-density lipoprotein and fibrocalcific deposits formation on aortic heart valves [16].

Besides, it has been shown that the lipid level reduction in the blood after pharmaceutical treatment with HMG-CoA reductase inhibitors (statins), which decreased the osteogenic pathways signaling, led to reduced aortic valve calcification in hypercholesterolemic animal models, compared with control, non-treated animals [16]. However, in contrary to this suggested mechanism, two different clinical trials using statins (Simvastatin and Ezetimibe) failed to show this inhibitory effect of statins regarding the progression of aortic valve calcification [17, 18]. The contradictory results between those different studies suggested that there are two possible explanations: (a) Statin anticalcification effect is a disease stage-dependent. Hence while it may help slowing down the progression of calcification in the later stage of the disease, it might not have the same effects in the early stages. (b)Statins have two opposing mechanisms concerning the calcification process. On the one hand, it works as HMG-CoA reductase inhibitors leading to reduction of calcification. On the other hand, statins have shown to induce osteogenic differentiation. Thus, in a valve where VIC have been differentiated into osteoblast-like cells, statins may have a procalcifying effect [10].

## Calcification of bioprosthetic heart valves

The pathogenesis of bioprosthetic heart valve calcification is even less understood than that of the native aortic valve. Still, the general mechanisms of both valves seem to be close [19].

Like in native valves, bioprosthetic calcification involves passive and active mechanisms. Concerning the active mechanism, there is a close similarity between both native and bioprosthetic heart valves like the valvular infiltration of cellular elements (smooth muscle cells, T-lymphocytes, monocyte macrophages, and mesenchymal cells) and the expression of noncollagenous matrix proteins [20]. The association of local cellular expression of noncollagenous matrix proteins with valvular calcification suggests that valvular mineralization may be an actively regulated process in both valve types.

Concerning the passive mechanism, calcification of the ECM structural proteins, collagen and elastin, has been reported in clinical and experimental bioprosthetic valves in a rat subdermal model. Usually, collagen and elastic fibers, provide the major active sites for nucleation and crystal growth of calcium-phosphate phases [21]. The extent of calcification is usually promoted by the usage of crosslinkers like e.g. glutaraldehyde and/or formaldehyde [22, 23]. In the native valve calcification mechanism (without crosslinking), the interaction between calcium (in the extracellular fluid) with phosphorus (in the cell membrane remnants) leads to primary nucleation of different calcium-phosphate crystals phases [22, 23]. Similarly, in the presence of crosslinkers, it was suggested that the disruption of calcium pumps in cell membrane increases, which causes a sharp increase of Ca^2+^ concentration inside the cells and raises the chance for further interaction with phosphate present at different sites of the cell (like cell membranes and DNA), thus forming the critical nuclei needed for the initiation of the process [22, 23]. Calcification proceeds and results in the formation of crystal aggregates appearing as nodules, which weaken the respective tissue and cause bioprostheses malfunction. Paradoxically, it has been shown that calcification is not a function of higher crosslinker concentrations. Glutaraldehyde at higher concentration of 3% in the fixation solution resulted in less calcification in comparison with the observed in the presence of 0.6% w/w concentration [21].

Another similarity between calcification in native and bioprosthetic heart valves is the effect of flexion points on heart valves calcification. The sites of intense mechanical deformations generated during the cardiac cycle in the heart valves (like the commissures and the base of the leaflets at the aortic ring) are the favorable positions for minerals deposition in both types [21].

## Native vs. bioprosthetic calcification

Recalling the possible calcification mechanisms presented in the previous sections, there is a lot in common between the native and bioprosthetic heart valve calcification processes. On the other hand, six main differences between those processes were identified and summarized in (Table 1).

**Table 1:** Six main differences between native and bioprosthetic heart valves

Parameter	Explanation	Ref.
Passive calcification mechanism	Both native and bioprosthetic heart valves involve passive calcification mechanisms. However, bioprosthetic valves, the effect of the debris of untreated dead cells is more pronounced.	[24]
The effect of the crosslinking agent “glutaraldehyde”	The presence of glutaraldehyde in bioprosthetic valves showed some procalcifying events: -Deactivation of membranes pumps allowing Ca^2+^ leakage from the mitochondrial membranes. -Increasing exposed areas through formation of void spaces. -Non-neutralized residues of glutaraldehyde. -Exposing larger numbers of more negative charges due to crosslinking of basic amino acids.	[27]
Bioprosthetic heart valve is accelerated version of native calcification	The calcification of native heart valves is a slowly proceeding disease. However, the bioprosthetic heart valves show a faster rate of progression in both children and elders.	[21]
Age prevalence of incidence	For non-pathological conditions, elders are under high risk of calcification of heart valves in comparison to children. In contrast, with bioprosthetic heart valves substitutes, the children would develop much faster calcification.	[28, 29]
Structural differences of calcific deposits	The solubility of mineral deposits of bioprosthetic heart valves is higher than in the corresponding deposits in the native valves.	[32]
Immunological consideration	Several immune responses have been involved in calcification of native heart valves. However, there is no sufficient evidences to support their role in calcification of bioprosthetic heart valves.	[21]

## Passive calcification mechanism

Even though calcification in both native and bioprosthetic heart valves, can proceed through the passive mechanism, it seems that there are some differences between the two classes of valves. In the case of bioprosthetic valves, the effect of the debris of untreated dead cells is more pronounced [24].

Advocates of the passive mechanism claim that the absence of cells in the calcific nodules, suggests a passive mechanism [20]. However, Demer argued that, in normal, regulated physiological mineralization such as bone growth, living cells are not normally present right at the nucleation site of calcium-phosphate at the beginning, but they are located at some further distance. Rather than directly secreting calcium-phosphate crystals, osteoblasts secrete a highly specialized extracellular matrix, which upon maturation acquires the architectural and physico-biochemical features needed to draw calcium and phosphate into the crystal phase. Cellular regulation, therefore, takes place at the level of matrix synthesis [25].

In the case of bioprostheses, there are not living cells during the time of implantation. Earlier suggestions that prosthetic valve tissue remains acellular in vivo are questionable since endogenous circulating cells may permeate the prosthetic valve matrix. For instance, endogenous myofibroblast-like cells invade and produce islands of collagenous tissue in the linings of cardiac assist devices 30 days after implantation [25]. Therefore, there is a valid possibility for the cells to infiltrate into the ECM of bioprostheses in vivo and to take part in the active calcification process.

Another criticism for the passive mechanism is the progression of calcification of porcine bioprosthetic heart valves upon implantation. Assuming that the main reason underlying calcification is the presence of calcifying potential of the original valvular tissue. This means they should calcify also in the animals from which they were extracted (e.g., pigs) [25]. However, they apparently do not calcify under normal physiological conditions. Two different possible explanations may be given: (a) Alteration of the type and structure of the ECM proteins caused by glutaraldehyde treatment; (b) Structural alterations in the lipids because of redox processes taking place [26]. It is suggested however that such oxidized lipids and cross-linked proteins are necessary but not sufficient for calcification [25].

To sum up, based on the previous argument, Demer et al. 1997 suggested that bioprosthetic valve calcification should not be considered as passive, but as endochondral calcification. In both cases, mineralization takes place in acellular tissues mainly at the stage of extracellular matrix organization and maturation [25].

## The effect of cross-linking agent

In native heart valves, there is no presence of crosslinkers (glutaraldehyde) contributing to the calcification mechanisms. On the other hand, the presence of glutaraldehyde in bioprosthetic heart valves is believed to contribute in four different ways in the calcification process [27]:

(a) In the passive calcification: it is taking place through the deactivation of membranes pumps (consequently damaging Ca–P electrochemical gradient across the membrane and allowing the Ca^2+^ leakage from the mitochondrial membranes, resulting to the formation of calcific deposits) [27];

(b) The formation of void space, resulting in the exposure of active sites or physical niche for calcification.

(c) Glutaraldehyde unreacted residues may promote calcification,

(d) Glutaraldehyde crosslinking with the basic amino acids in collagen helices, thus impairing charges balances and exposing larger numbers of more negative charges which may provide active sites for Ca^2+^ binding and hence with the concomitant development of nucleation sites for calcification.

Thus, in contrary to those bioprostheses, the native heart valves do not have any glutaraldehyde. Hence, all the possible effects can only take place in the bioprostheses HV.

## The bioprosthetic heart valve is an accelerated version of native heart calcification

According to Demer, calcific aortic valve disease is a variable severity disorder, advancing at a relatively low rate. It may be divided into two main stages: (a) Aortic sclerosis, involves mild valve thickening, in which there is no blood flow obstruction. (b) Aortic stenosis, accompanied by severe calcification, because of which the movement of leaflets is impaired. In general, the rates of bioprosthetic valve calcification are significantly higher in comparison with the respective process of native valves calcification [25]. It should also be noted that the rate of progression of calcification is markedly accelerated in younger patients. Children and adolescents have an especially accelerated course while elderly patients demonstrate a lower rate of bioprosthetic valve degeneration [21].

## Age prevalence of incidence

As in native aortic valves, calcific changes in bioprosthetic valves is a prominent feature of primary valve failure. However, the prevalence of calcification and the bioprosthetic valve failure appears to decrease with the age in contrast to native valves. In several studies, younger ages were more vulnerable to a bioprosthetic valve failure and a need for reoperation [28, 29]. This finding indicated that the calcification process of bioprosthetic valves may be different from the observed in native valves [29]. The possible explanation for such a correlation is that remodeling rate is different between children and adults, In young ages, high rate of remodeling is accompanied with the differentiation of VIC into myofibroblast due to chronic stress [30]. Those myofibroblast express osteoblast markers (osteocalcin and bone sialoprotein), leading finally to the formation of nodules-like bone-type calcific deposits inside the valvular tissue structure [31].

## The structural differences of calcific deposits

Physicochemical characterization of the calcific deposits with the powder X-ray diffraction and Fourier Transform Infrared spectroscopy showed that the crystalline phases of human heart valves calcific deposits collected from different pathological cases, were similar in terms of chemical composition, crystal structure, morphology and solubility [32, 33]. However, the solubility of mineral deposits of bioprosthetic heart valves was higher in the corresponding deposits on native valves [32]. A possible explanation for this difference may be due to shorter residence and ageing time of the mineral deposits in the bioprosthetic valves, including transient crystal phases, which eventually dissolve easier compared to more stable phases formed in the native ones [34]. Further confirmations for this possibility came from the finding that the structure of the calcific deposits developed on bioprosthetic valves are less crystalline, in comparison with the corresponding formations on the natural heart valves deposits [32].

## Immunologic consideration

The role of immunological responses in the heart valve calcification is a very controversial and broad topic. As mentioned in the previous sections, several immune responses have been involved in calcification of native heart valves. However, Schoen and Levy argued the sufficiency of the pieces of evidence for the role of the immune system in the calcification of bioprosthetic heart valves as follows; (a) animal immunological sensitization is not only limited to fresh tissues but also cross-linked tissues, (b) in different valve dysfunction disorders, antibodies were detected after, not before the dysfunctionality, (c) very often mononuclear inflammatory cells were found in the failed tissues valves. However, this finding is not enough to consider it as immunologic rejection. Accordingly, there is no solid evidence for the hypothesis of the role of inflammatory cells for heart valve calcification processes. This opinion was further supported by the finding that there was no difference in calcification potential of the heart valve cusps between groups, which were enclosed in a filter chamber (isolated from host cells contact) and a control group [21].

Despite the six mentioned differences stated above between native and bioprosthetic heart valves, comparison between native and bioprosthetic calcification is a quite challenging issue. The reason for such difficulty is attributed to the wide variety of bioprosthetic valves with each of them having their own characteristics. However, literature reports mostly focus on the glutaraldehyde-treated porcine or glutaraldehyde-treated bovine pericardial valves.

## Calcification of decellularized heart valves

Although bioprosthetic heart valves present a better hemodynamic profile and anticoagulation free post-implantation lifespan, at the present, calcification restricts the patients’ profile to elderly or people noncompatible with anticoagulation therapies (e.g., pregnant women) [35]. Moreover, considering the previously mentioned drawbacks, associated with different types of heart valves bioprostheses, a new class of substitutes emerged, known as TEHV. The idea behind this new type is to use them as temporary scaffolds, while in parallel to restore the normal function of the native valve, enhance host cells infiltration in vivo and support their response to mechano-transduction to regenerate normal live valvular tissue [8]. Upon proceeding of this new tissue, the scaffold material is expected to be gradually biodegraded until its total replacement by the generated new living tissue valve. Among different types of TEHV, the decellularized ones (dTEHV) have attracted the interest, because of their low cost, simple preparation protocols and their similarity to the native valves. Till 2020 more than 80 articles and reviews have introduced and evaluated the use of these dTEHV [36–44].

Despite the relatively poor knowledge of calcification possible mechanisms in those types of heart valves, several research groups pointed to the role of immunogenicity and cell remnants as the main culprits in the calcification [10]. Hence the decellularization process is expected to reduce or eliminate the calcification.

So far, many animal and clinical studies have been published since 2000, in which decellularized heart valves, were used for the replacement of defective ones (Tables 2 and 4). Up to present, there is no comprehensive review representing and discussing those data in the light of their calcification potential.

**Table 2:** Animal studies conducted to date with decellularized heart valves and their correlation to calcification

Calcification prevention	Xenogeneic/Allogeneic	Graft Source	Decellularization Technique	Cells	Model	Max. Implantation duration	sample size	Characterization	Aortic/Pulmonary	Ref.
Yes	Xenogeneic Allogeneic	Pig Sheep	Chemical/Enzymatic SynerGraft Tech (Hypotonic, RNAse, DNase)	Unseeded	Sheep	11 m	9 Pig 4 Sheep	Gross examination	A & P	[84]
Yes	Xenogeneic	Pig	Chemical 24 h in 0.1%DOA, then stored in Hanks buffer with antibiotics	Vascular Ecs	Juvenile sheep	8 m	8	X-ray; flame atomic absorption spectrometry,Von Kossa	P	[85]
Yes	Xenogeneic	Pig	Chemical (AutoTissue Ltd) 1%DOA/ 70% EtOH, then kept in RPMI nutrient medium up to 30 days before implantation	Unseeded	Juvenile sheep	11 m	4	X-ray; flame atomic absorption spectrometry, Von Kossa; gross exam	P	[58]
Yes	Allogeneic	Sheep	Chemical/Enzymatic For 24 h tissues were treated with a solution of 0.5% SOC and 0.5% SDS for 24 h. Then for 72 h (6 cycles) with PBS supplemented. Finally, tissues were treated by a DNase for 4 h at 37 °C	12 ECs 9 Unseeded	Merino lambs	3 m	21	Von Kossa staining, gross examination	P	[64]
Yes	Xenogeneic	Pig	Chemical Valves were treated for 14 h with 1% DOA in physiological saline at 37 °C then washed with physiological saline.	Unseeded	Juvenile sheep	2 y	11	ECG	P	[59]
Yes	Xenogeneic	Pig	Chemical/Physical valves were immersed in PEG solution containing antibiotics for 168 h under stirrer. The PEG solution was changed every 48 h. The valves were exposed to 100 kGy gamma irradiations in room air. The valves were washed in normal saline solution for 24 h, then transferred into DNase solution for 48 h at 37 °C. The DNase solution then valves were washed with normal saline solution for 3 h.	Unseeded	Rats Dogs	Rats: 2 m Dogs: 6 m	20 Rats 9 Dogs	Von Kossa staining	A	[63]
Yes	Allogeneic	Sheep	Chemical Valves were decellularized at room temperature in a solution of 0.5% SDC and 0.5% SDS for 48 h, followed by 2 wash cycles (12 h each) in distilled water, then six wash cycles with PBS under continuous shaking. Then valves were stored in PBS at 4 C for one day.	Unseeded	Sheep	9 m	12	Von Kossa staining; Gross examination	A	[55]
Yes	Allogeneic	Sheep	Chemical/Physical Before decellularization valves were kept in RPMI 1640 containing 10% DMSO and 10% FBS and cryopreserved for 48 h. At room temperature, tissues were treated with an N-lauroyl sarcosinate in a Tris buffer solution for 24 h containing a recombinant endonuclease and antibiotics. Then the tissues were rinsed by recirculating water through a bed of resin for 24 h. The valves were divided into 2 groups: 1- No further processing and placed into isotonic saline with polymyxin B then stored at 1 °C to10 °C 2- Glycerolized for 24 h then stored at −80 °C	Unseeded	Juvenile sheep	1 y	1) 5 2) 5	Gross examination; HR-X-ray; EGG; MRI; Angiography	p	[68]
Yes	Allogeneic	Sheep	Chemical Firstly, tissues were immersed in a RMPI 1640 solution containing antibiotics for 24 h at 4 °C. Then, decellularized was done with a solution called PUC I (Brazilian patent), consisting mainly of 0.1% SDS under shaking. Decellularized tissues were preserved in RPMI 1640 solution containing antibiotics at 4 °C.	Unseeded	Juvenile sheep	6 m	8	Gross examination; ECG; Alizarin Red	P	[86]
Yes	Allogeneic	Pig	Chemical DOA	Unseeded	Pigs	6 m	12	Gross examination; HR-X-ray; Von Kossa	A	[87]
Yes	Allogeneic	Sheep	Chemical/Enzymatic After 72 h of cold ischemia, valves were decellularized using 2 anionic, nondenaturing detergents (N-lauroylsarcosine and Triton-X, different osmolality solutions, endonuclease, and ethanol (40% v/v). Valves were washed for 24 h with deionized water in the presence of ion exchange resins at 21 °C. Finally stored at 4 C in an antibiotic solution.	Unseeded	Juvenile sheep	5 m	8	Gross examination; X-ray; Von Kossa	P	[62]
Yes	Allogeneic	Sheep	Chemical Valves were decellularized at room temperature in a solution of 0.5% SDC and 0.5% SDS for 48 h, followed by 2 wash cycles (12 h each) in distilled water, then six wash cycles with PBS under continuous shaking. Then valves were stored in PBS at 4 °C for one day	Jugular vein ECs	Sheep	3 m	5	Gross examination; HR-X-ray; EGG; MRI; Von Kossa	A	[69]
Yes	Allogeneic	Sheep	Chemical/Enzymatic For 24 h, the valves were immersed in a wash solution (20 mM EDTA, 0.3% NaCl, protease inhibitor cocktail and 0.05% NaN3) for at 4 °C. For 144 h (with daily renewal), the solution was replaced with another one containing 0.5% SDS, 0.5% Triton X-100 in a 0.3% NaCl solution containing 0.05% NaN3 for under shaking. Finally, the valves were washed for 144 h (with renewal every 3 h) with a solution of 0.3% NaCl solution containing 0.05% NaN3 under shaking. The valves were stored at room temperature in a solution of 200 ml/valve, 0.6% NaCl, 20 mM EDTA, 10 mM HEPES and 0.05% NaN3.	Unseeded	Sheep	5 m	14	Gross examination, X-ray; ECG; spectrophotometry	A	[54]
Yes	Allogeneic	Pig	Chemical/Enzymatic “TRICOL” Tissues decellularized was done using b hypo- or hypertonic solutions, Triton X100 and SDC. Later, detergents, then endonuclease was used.	Unseeded	Pig	15 m	22	Gross examination; Von Kossa	A	[60]
In-between	Xenogeneic	-Pigs -Kangaroos	Chemical/Enzymatic -Tissues were treated with a hypotonic Tris-buffered (pH 8.0) followed by a hypertonic NaCl solution containing PMSF, protease inhibitor, and antibiotics. later tissue was treated with enzymatic solutions of DNase, RNase, trypsin protease, and phospholipases). Finally, the tissues were washed in a Ca & Mg-free chelating solution. -	Unseeded	Sheep	120 d	−3 −3	Von Kosa, SEM, Ashing and weighing the Toronto SPV valve leaflets (glutaraldehyde fixed commercial product) showed amount of calcium (dry weight) 2.63 mg/g compared to both decellularized tissues kangaroo (43.81 mg/g) and porcine (105.08 mg/g).	A	[75]
In-between	Allogeneic	Sheep	Enzymatic/ chemical For 24 h, the tissues were treated with a PBS solution containing 0.05% trypsin and 0.02% EDTA at 37 °C under shaking. The valves were washed with PBS then stored in HBSS at 4 °C.	ECs, myofibroblasts	Sheep	3 m	10	Gross examination; histology Discrete subvalvular calcifications were observed. No valvar calcifications were found.	P	[9]
In-between	Allogeneic Xenogeneic	Sheep Pig	Enzymatic/Chemical The tissues were treated with 0.05% trypsin and 0.02% EDTA for 48 h, then washed for 2 days with PBS	Unseeded	Sheep	6 m	3 Sheep 3 Pigs	Gross examination; Von Kossa After 12 weeks, all AVMC showed severe calcification while after 24 weeks, XVMC showed only mild calcification.	P	[73]
In-between	Xenogeneic	Pig	Enzymatic/Chemical Four different treatment were used 1)Trypsin At 37 °C, the valves were placed in a solution of 0.05% trypsin and 0.02% EDTA (pH 8.0) at for 4 h under continuous shaking. 2) Osmotic treatment At 4 °C tissues were treated with a hypotonic solution (10mM Trizma) followed by 10mM Trizma HCl (each for 48 h). Both solutions contained 5mM EDTA, pH 8.0 and were done under gentle shaking. 3) Trypsin-osmotic treatment At 4 °C tissues were treated with a hypotonic solution Tris-buffer (pH 8.0), then a hypertonic 0.5% Triton X-100 solution (each for 2 days) under gentle shaking 4) Detergent-osmotic shock At 4 °C tissues were treated with a hypotonic solution Tris-buffer (pH 8.0), then a hypertonic 0.5% Triton X-100 solution (each for 2 days) under gentle shaking. Finally, for all those protocols’ tissues were washed for 3 days with PBS under shaking.	Unseeded	Rat	1w	4 Pigs 4 Pigs 4 Pigs 4 pigs	Von Kossa; TEM Only first group of decellularization (trypsin alone) showed calcification after one week.	A	[49]
In-between	Allografts	Sheep	Chemical/Enzymatic For 24 h tissues were treated with a solution of 0.5% SOC and 0.5% SDS for 24 h. Then for 72 h (6 cycles) with PBS supplemented. Finally, tissues were treated by a DNase for 4 h at 37 °C	CCN1-coated/ autologous endothelial-like cells (EC)	sheep	12 m	Sheep	Van Kossa Only 2 out of 17 showed calcification.	P	[88]
In between	Allografts	Pig	Chemical/Physical For 48 h, valves were treated with a solution containing 0.5% SDC and 0.5% under continuous shaking then -for 72 h rinsed by a 72-h rinsing step using demineralized water. Finally, a succession of baths of acetone, ethanol, NaOH and Hydrogen Peroxide. Finally, the tissues were kept at −80 °C without solution.	unseeded	Pig	3 m	Pig	Von Kossa staining Only one animal showed minimal calcification after 3 months.	P	[89]
No mention	Allogeneic Xenogeneic	Sheep Pig	Enzymatic 48-h enzyme incubation was done. (what enzyme?)	Myofibroblasts; ECs	Sheep	9 m	15	–	P	[65]
No mention	Allogeneic Syngeneic	Rat	Chemical/ Enzymatic Valves were treated with 4 different protocols were use: 1-Triton X-100 (1–5%) for 24 h 2-Trypsin (0.5%) for 0.5–1.5 h 3- 0.5 Trypsin for 0.5–1.5 h followed by Triton X-100 (/1–5%) for 24 h 4-SDS (0.1–1%) for 24 h followed by DNase and RNAse treatment, then washed by HBBS for 48 h at 4 °C.	Unseeded	Rat	21d	4	–	A	[56]
No mention	Xenogeneic	Pig	Enzymatic/Chemical The tissues were decellularized using 0.05% trypsin and 0.02% EDTA for 48 h, followed by rinsing with PBS solution for 48 h under mild shaking at 37 °C.	Myofibroblasts & ECs	Sheep	6 m	8	–	P	[90]
No mention	Allogeneic Syngeneic	Rat	Chemical/Enzymatic Firstly, Samples were placed in hypotonic Tris buffer (10 mM, pH 8.0) containing PMSF 0.1 mM and EDTA 5 mM for 48 h at 4 °C. Then tissues were transferred to Secondly, samples were placed in 0.5% Triton X-100 in a hypertonic Tris-buffered solution (50 mM, pH 8.0; PMSF, 0.1 mM; EDTA, 5 mM; KCl, 1.5 M) for 48 h at 4 °C. Samples were then rinsed with Sorensen’s phosphate buffer (pH 7.3) and placed in Sorensen’s buffer containing DNase, RNase and MgCl2 for 5 h at 37 °C. Thirdly, Samples were placed in Tris buffer (50 mM, pH 9.0; Triton X-100 0.5%) for 48 h at 4 °C. Finally, all samples were washed with PBS at 4 °C for 72 h (renewal every 24 h). All steps under continuous stirring.	Unseeded	Rat	16 w	6	–	A	[91]
No mention	Allogeneic	Rat	Chemical/Enzymatic Three different protocols were used: 1- Firstly, Samples were placed in hypotonic Tris buffer (10 mM, pH 8.0) containing PMSF 0.1 mM and EDTA 5 mM for 48 h at 4 °C. Then tissues were transferred to Secondly, samples were placed in 0.5% Triton X-100 in a hypertonic Tris-buffered solution (50 mM, pH 8.0; PMSF, 0.1 mM; EDTA, 5 mM; KCl, 1.5 M) for 48 h at 4 °C. Samples were then rinsed with Sorensen’s phosphate buffer (pH 7.3) and placed in Sorensen’s buffer containing DNase, RNase and MgCl_2_ for 5 h at 37 °C. Thirdly, Samples were placed in Tris buffer (50 mM, pH 9.0; Triton X-100 0.5%) for 48 h at 4 °C. Finally, all samples were washed with PBS at 4 °C for 72 h (renewal every 24 h). All steps under continuous stirring. 2-The same of (1) except there is no Triton-100X 3- Valves were decellularized with solution of PBS containing Trypsin 0.5%/EDTA 0.2 at 37 °C for 48 h (renewal twice) under shaking.	Unseeded	Rat	1 w	18	–	A	[92]
No mention	Xenogeneic	Pig	Enzymatic/chemical 4 different protocols were used: 1- were treated with 1% SDC in PBS at 37 C for 24 h 2–1% SDS in PBS at 37 C for 24 h 3–0.05% trypsin /0.02% EDTA in PBS at 37 °C for 24 h 4–0.1% trypsin/0.02% EDTA in PBS at 37 °C for 1 h, then transferred to hypotonic 0.01 M Tris buffer pH8.0 containing PMSF at 4 °C for 4 h, later hypertonic 0.05 M Tris buffer pH8.0 containing Triton X-100 and PMSF at 4 °C for 4 h, finally, DNase /RNase incubating at 37 °C for 2 h, with hypertonic Tris buffer at 4 °C for 4 h.	ECs, myofibroblasts	Sheep	4 w	3	–	P	[66]
No mention	Xenogeneic	Human	Chemical/Enzymatic At 37 °C the tissues were kept overnight in PBS containing 0.25% Triton X-100, 0.25% SDS, 0.02% EDTA then washed twice with PBS. Different tissues were kept in different Benzonase solutions (100,80,20 U/ml) of 50-mM TRIS-HCI buffer, pH 8.0, supplemented 1 mmol/l of MgCl_2_ at 37 °C with different durations. with a nuclease digestion solution supplemented with 80 Several washing solutions were utilized PBS, M-199 medium sequentially for more than 24 h at 4 °C. All steps were done under shaking. The tissues were stored in fresh M-199 at 4 °C.	Human vascular ECs	Baboons	8w	8	–	P	[93]

*DOA* deoxycholic acid, *SDS* sodium dodecyl sulfate, *EDTA* ethylenediaminetetraacetic acid, *RNAse* ribonuclease, *DNase* deoxyribonuclease, *EtOH* Ethanol, *PEG* polyethylene glycol, *SDC* sodium deoxycholate, *DMSO* dimethyl sulfoxide, *FBS* fetal bovine serum, *PMSF* phenylmethylsulphonyl fluoride, *BHA* butylated hydroxyaniso, *AVMC* acellularized allogenic ovine valve matrix conduits, *XVMC*: acellularized allogenic xenogeneic porcine (XVMC) valve matrix conduits

In the present review, we focus mainly on the calcification potential of those implanted dTEHV scaffolds from both animal and human heart valves through clinical and animal studies. pericardial tissue-based decellularized heart valves [45] and decellularized synthetic-seeded scaffolds [46] will not be covered in this review.

## The effect of the decellularization on the calcification from animal studies

Since the emergence of tissue decellularization concept as a promising option for s+caffolds preparation in the tissue engineering field, wide variations of techniques have been utilized. The purpose of all those techniques is always the same:

(1) Preservation of the structural integrity of the original tissue ECM; to restore its mechanical and biological properties.

(2) Preservation of and/or enrichment with important growth factors.

(3) Removal of any cell debris and any immune triggering parts.

Theoretically, based on the calcification possible mechanisms discussed earlier, successful decellularization would prevent calcification without a need for any further treatment.

In general, the history of acellular tissue in cardiovascular application backs to the work of Vesely in vitro study in 1992, regarding in vitro studies [8, 47].

Since then, many animal studies have been conducted on decellularized tissues, in which different kinds of animal models (Sullfok Sheep, Dog, Rats, Kangaroo, Pigs, chacma baboons, merino lamb), immunotype of the used heart valves (Xenografts, Homografts and Allografts) and decellularization methods (Enzymatic, detergents, combined), cell-seeded or unseeded have been used. The mineralization process was monitored using different characterization tools separately or together (Gross examination, Echocardiography, MRI, Electron Microscopy, Atomic absorption, and Histology) (Table 2).

The first published in vivo study for the evaluation of decellularized heart valves was by O’Brien and colleagues in 1999. In this work, porcine aortic valves were decellularized by a process designed to remove all the leaflet cells. The treated valves were next implanted in weanling sheep. After 150 days, the grafts were explanted and assessed histologically and analyzed for calcium content by the atomic absorption spectrometry. All valves were hemodynamically functional at explant. Histological examination of the explanted valvular tissues showed structurally intact leaflets with in-growth of host fibroblastoid cells without evidence of calcification. The calcium content in porcine leaflets was unaltered over the duration of the implant. The lack of calcification of acellular aortic leaflets suggested that prolonged durability of such valves is attainable, without the use of cross-linking agents [48].

Since then, large numbers of animal and clinical studies have been published. In this review, those animal studies are sorted and further subcategorized into four different categories (Tables 2 and 4) for each, depending on the effect of decellularization on calcification: (A) Decellularization totally prevented calcification, (B) Decellularization reduced calcification, (C) Decellularization showed both pro-calcification and anticalcification potential based on the conditions involved and finally (D) Studies did not mention clearly the effect of decellularization on calcification. All of those categorized were tabulated and compared to each other’s depending on the host animal (in the case of animal studies), the type of heart valves involved, the technique of decellularization, cell seeding or not and type of cells used, duration of the study follow-up, the size of the group involved and finally the techniques used to evaluate calcification occurrence). Also, so far nearly around five specific articles comparing different decellularization techniques effect on calcification have been published; in vivo studies [49–51] and in vitro studies [52, 53].

### Categories A&B: decellularization results in calcification reduction or prevention

Different approaches showed a successful anticalcification effect of the used decellularization techniques: Some of them are without any further post-decellularization process (only decellularization) as SDS [54], SDS/osmotic shock [42, 55], SDS/ RNAse/DNase [56], DOA [57], DOA/EtOH [58, 59], DOA/triton x-100/endonucleases [60], N-lauroylsarcosine/cryo [61], N-lauroylsarcosine/Triton-X/EtOH [62], PEG/gamma radiation [63].

Other reports had post-decellularization treatment as cell seeding (endothelial cells [57, 64], endothelial and myofibroblast cells [65, 66]), proteins and/or growth factors treatments as fibronectin [57, 67], and fibronectin/SDF-1 [54] have been used. Although both treatments improved coverage of the decellularized tissue with endothelial cells, they showed no difference regarding anticalcification treatment (both treated and untreated decellularized ovine aortic allograft did not calcify).

Different post-decellularized preservations also have been investigated like cryopreservation [62, 68, 69]. According to those three publications, a clear anticalcification effect of the decellularized group in comparison to bioprosthetic valves (either xenografts or allografts) has been shown.

Noteworthy, in this category (A&B) the slight formation—in some cases—of calcific deposits were attributed to the immune response of the injuries related to suturing. Therefore, the use of sutureless or transcatheter would—theoretically—overcome this problem. Some animal studies showed promising results for using percutaneous dTEHV implantation (no calcification and good remodeling) [70]. On the other hand, a report showed a different complicated scenario when evaluating the effect of this technique on the calcification on general. This study discussed the effect of the severity and distribution pattern of calcific deposits of heart valves on the percutaneous implantation success [71].

### Category C: decellularization results in conditional calcification

**Fig. 2:**
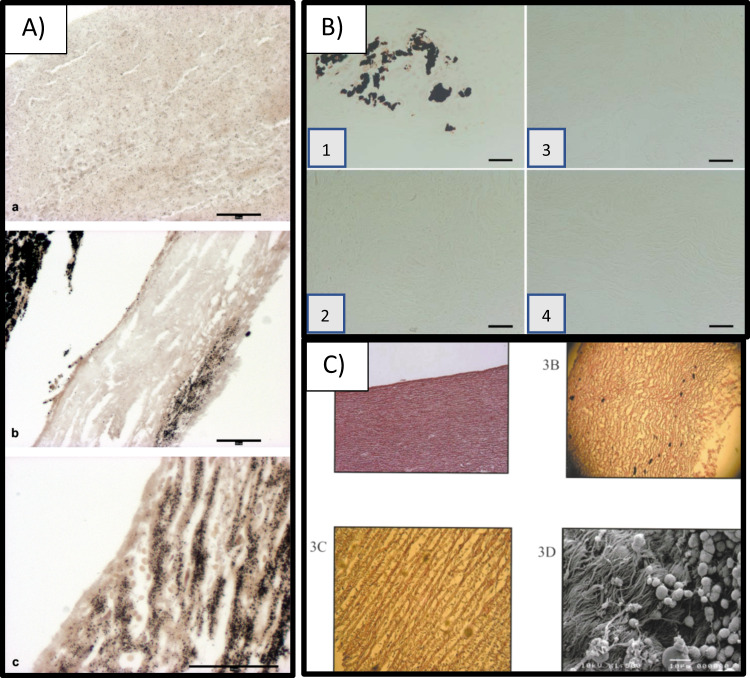
Different calcification modes in decellularized heart valves: **A** Von Kossa stain showed comparison between SPV Glu-fixed heart valves (upper image), porcine matrix (middle) and decellularized Kangroo matrix showing lowest calcification in the SPV samples for calcium in explant [75], **B** Von Kossa staining of (B-1) trypsin, (B-2) osmotic, (B-3) trypsin-osmotic, and (B-4) detergent-osmotic treated matrices seven days after implantation. Calcific deposits are only present in trypsin matrices (B-1), as indicated by black staining [49], **C** Pre-implantation histologic for model 700 SynerGrafte heart valves showed multiple calcific deposits within the matrix [81]

In contrary to the reports that showed the absence or just suture line calcification, few ones showed that decellularization would not prevent calcification (Fig. 2).

For example, Steinhoff and associates showed that decellularized allogeneic heart valves implanted in sheep model demonstrated calcification after using EDTA/trypsin decellularization technique with autologous endothelial and myofibroblast. The calcification mainly took place around the cell remnants [9]. It was also associated with a strong leukocyte infiltration early after implantation, suggesting that an inflammatory reaction was responsible for the underlying mechanism of early-stage calcification. This suggested mechanism is with an agreement with a previously published explanation of calcification in blood vessel’s wall by Andrews et al. [72]. They suggested that the wall would simulate the generation of activated platelet aggregates or thrombus acceleration, because of glycoprotein binding availability in collagen. The adhered and activated platelets might also interact with inflammatory leukocytes as well, facilitating leukocyte-endothelial cell adhesion, leading finally to calcification [72]. The conclusions from this work further corroborate the suggestion of an immuno-mediated calcification [9]. It is noteworthy here to mention that the sensitivity response for any injuries between different animals’ models is an important factor when studying calcification, as showed that sheep injuries typically respond with calcification (i.e. more sensitive to calcification than human) [68].

In another report decellularized Xeno- and allograft implantation using the same decellularization technique without any cell seeding were compared [73]. Surprisingly, calcification was detected on the allograft only. Careful examination of both scaffolds showed that XVMC (xenogeneic acellular valve matrix conduits) had a higher number of recruited cells with wide variations of cell types. The interior of the xenogeneic (porcine) matrix was repopulated with all three vascular cell types (endothelial, myocytes, and fibroblasts) in approximately natural proportion, while the surface of XVMC was partially populated with endothelial cells only. On the other hand, the surface of the allogeneic (ovine) matrix was also partially repopulated with endothelial cells only, while the interior of the matrix was dominated with fibroblasts alone. The authors assumed that there was an initial full cell repopulation upon implantation for both scaffolds. However, for some reasons, myocytes or other cells started dying in AVMC (allogenic acellular valve matrix conduits), because of some factors related to the micro-environment of this scaffold. The domination of fibroblasts within AVMC, supported this hypothesis because it is known that fibroblasts are more resistant to micro-environmental changes. Since in both cases, the same decellularization techniques were used, the observed differences could be attributed to a significant extent to specific differences in ECM composition between different species, especially on that of the native pulmonary valve. Again, the further semiquantitative analysis showed a difference in composition of ECM between AVMV and XVMC, with higher amounts of glycosaminoglycans and proteoglycans in the AVMC [73]. Regarding AVMC, extensive work has shown no calcification for the decellularized scaffolds after in vitro dynamic reendothelialization and conditioning [74].

Van Nooten et al., using detergents/enzymes combinations, showed that calcification in the decellularized Xeno- and allograft was higher than in commercially available glutaraldehyde-treated porcine heart valve (SPV, Toronto) [75].

In another report, the same author compared different decellularization techniques: trypsin alone, trypsin-osmotic shock and detergent-osmotic shock. Calcification was only noticed in the trypsin-treated group [49]. Immunogenicity and calcification potential of trypsin and trypsin-osmotic pressure-treated ovine valvular tissue matrices were compared in vivo using a rat model. It was shown that a strong CD3 - T-cell inflammatory infiltration was accompanied with calcific deposits after 1 week in the trypsin-treated matrices. This was not identified on any of trypsin-osmotic treated leaflet matrix. A possible explanation could be that the collagenous matrix of trypsin-treated implants was probably more damaged in comparison with trypsin-osmotic pressure-treated leaflets. Mineralization of biological valves usually begins at the sites of disrupted collagen fibers in the damaged ECM (where VIC are activated to take part in the natural remodeling procedure). It seems that their activation and function is related to the calcification mechanism [76]. The loss of GAGs (Glycosaminoglycans) in these scaffolds was not responsible for calcification. It was previously speculated that the presence of negatively charged GAG molecules within the ECM of cuspal tissue may reduce calcification, by chelating calcium ions, thereby reducing supersaturation and preventing hydroxyapatite nucleation and growth. However, in this study, the GAG-depleted trypsin-osmotic matrices did not form calcific deposits [49].

Finally, the presence of decellularizing agent remnants (e.g., DOA) after completion of the decellularization process would contribute to the formation of calcific deposits through the passive mechanism. In such a case, the inefficient washing techniques after decellularization is a sufficient cause to calcification [52].

Based on the previous reports, the causes of calcification in the decellularized heart valves can be classified into four main reasons (Table 3).

**Table 3:** Causes of the calcification in decellularized heart valves possible

Possible cause	Possible explanation	Reference
Damaged ECM	Mineralization of biological valves usually begins at the sites of disrupted collagen fibers. (why)	[49]
Immunological response	It takes place against immune triggering materials like cell remnants in inefficient decellularization, death of implanted or in vivo recruited cells or in the presence of α-gal (in porcine valves). Usually, immunological events, differentiation and trans-differentiation took place.	[9, 68, 72, 94]
Species individual differences of extracellular matrix composition	Heart valves from different species has different ECM structure and components which influence their interaction with different types of host cells.	[64, 73]
Residual of detergent	Under physiological conditions some decellularizing agents like deoxycholic acids residues may enhance the formation of calcific deposits inside ECM.	[52]

## The effect of the decellularization on the calcification from clinical studies

Since 2002 till now (2020), around 30 publications and four main reviews [39, 42, 77, 78] have been published regarding decellularized heart valves implantation in humans. Some of them are case studies, while others are clinical trials.

The first published study was done by Dohmen et al., reported that a 43-year-old patient suffering from aortic valve stenosis underwent a Ross operation using pulmonary cryopreserved allograft (following DOA decellularization and in vitro EC seeding) to reconstruct the RVOT (right ventricular outflow tract). Multislice computed tomography examination showed that there were no calcifications in both heart valves after 1 year [79].

The diversity of the different techniques used for decellularization, cell seeding, and additional treatment are very similar to the one that took place in animal studies, as presented in Table 4.

**Table 4:** Clinical studies conducted to date with decellularized heart valves and their correlation to calcification

Prevent calcification	Xenogeneic/Allogeneic	Graft source	Decellularization technique	Cells	Max. implantation duration	Sample size	Characterization	Aortic/Pulmonary	Ref.
Yes	Allogeneic	Human	Chemical At 37 °C, the valves were treated with 1% DOA, followed by an extensive wash in normal saline.	Unseeded	1 y	1 Case	MRI; ECG	P	[79]
Yes	Allogeneic	Human	Synergraft	Unseeded	15 m	22	ECG	A	[95]
Yes	Allogeneic Xenogeneic	Human Pig	1) Cryolife 2) Autotissue	ECs	3 y	1) 11 2) 12	ECG; MRI	P	[96]
Yes	Allogeneic	Human	Chemical For 10 days, the valves were treated with SDS, and stored in medium RPMI medium at 4 °C	Unseeded	25 m	51	ECG	A	[97]
Yes	Allogeneic	Human	SynerGraft	Unseeded	5y	342	ECG	P	[82]
Yes	Allogeneic	Human	1- Chemical Two different protocols were used: The tissues were treated with DOA 2- The valves were treated with SDS	1) Unseeded 2) ECs	11y	1) 39 2) 44	ECG	P	[98]
Yes	Allogeneic	Human	Chemical Tissues were treated with SDS 0.1% for 24 h under continuous shaking, then washed for 10 days with Ringer Lactate solution	Unseeded	5y	41	ECG; MRI; CT	A	[99]
Yes	Xenogeneic	Pig	Matrix P plus	Unseeded	17 m	16	ECG	P	[100]
Yes	Allogeneic	Human	AutoTissue	ECs	10 y	11	ECG; MRI	P	[101]
Yes	Allogeneic	Human	Chemical At room temperature, tissues were treated with 0.5% SDC and 0.5% SDS for 36 h. Finally, Tissues were washed with NaCl 0.9% solution and stored at 4 °C	Unseeded	5 y	17	MRI; ECG	P	[102]
Yes	Homograft	Human	ESPOIR	unseeded	7 y	5	CT	P	[103]
No	Allogeneic	Human	Synergraft	Unseeded	2 y	1	IHC; Gross examination	A	[104]
No	Xenogeneic	Pig	Matrix P plus	Unseeded	1 y	106	IHC	P	[105]
No	Allogeneic	Human	Synergraft	Unseeded	1 y	4	ECG; Gross examination; Histo; SEM	P	[81]
No mention	Allogeneic	Human	SynerGaft	Unseeded	3 y	41	-ECG	P	[106]
No mention	Allogeneic	Human	Chemical/ Enzymatic First tissues were treated with hypotonic solutions then by enzymatic solution containing RNAse/ DNase.	Unseeded	5 y	47	-ECG	P	[107]
No mention	Allogeneic	Human	AutoTissue	Unseeded	18 m	11	–	P	[58]
No mention	Allogeneic	Human	Synergraft	Unseeded	19 m	26	–	P	[108]
No mention	Allogeneic	Human	Enzymatic/Chemical For 48 h, tissues were treated with a PBS solution containing 0.5% Trypsin and 0.2% EDTA at 37 °C (2 cycles).	EPCs	40 m	2	–	P	[109]
No mention	Allogeneic	Human	Chemical Two different protocols were used: 1- (AutoTissue®) The tissues were treated with DOA, 1%, and ethanol, 80% 2- The tissues were treated with SDS, 0.1%.	Unseeded	4 y	1)35 2)33	-	P	[110]
No mention	Xenogeneic	Pig	Matrix P plus	Unseeded	15 m	11	–	P	[111]
No mention	Allogeneic	Human	SynerGraft	Unseeded	9 y	29	–	P	[112]
No mention	Allogeneic	Human	SynerGraft	Unseeded	10 y	39	–	P	[113]
No mention	Allogeneic	Human	Chemical The tissues were treated with SDS 0.1%	Unseeded	3 m	6	–	P & A	[114]
No mention	Allogeneic	Human	Chemical At room temperature, tissues were treated with a solution of 0.5% SDC and 0.5% SDS for 48 h, then washed with distilled water 24 h, 12 h each followed by two washing process in distilled water, and PBS. All steps were done under shaking. Finally, tissues were stored in PBS at 4 °C.	Unseeded	3 y	47	–	P	[69]
No mention	Allograft	Human	ESPOIR	Unseeded	3 y	6 cases	–	P	[115]
No mention	homograft	Human	Chemical At room temperature, tissues were treated with 0.5% SDC and 0.5% SDS for 36 h. Finally, Tissues were washed with NaCl 0.9% solution and stored at 4 °C	unseeded	10 y		–	P	[83]

*DOA* deoxycholic acid, *SDS* sodium dodecyl sulfate, *EDTA* ethylenediaminetetraacetic acid, *RNAse* ribonuclease, *DNase* deoxyribonuclease, *EtOH* Ethanol, *PEG* polyethylene glycol

Based on the previous table results, most of those clinical studies showed a promising anticalification effect upon decellularization of the heart valves.

## Commercial products

To the best of our knowledge, there are two main products of decellularized heart valves and one from decellularized porcine sheets (Table 5) [80].

**Table 5:** Commercially available decellularized heart valve products and their calcification potential

Commercial name	Company	Source of valve	Calcification potential	Ref
CryoValve Synergraft® family (Aortic valves and SG pulmonary valves)	CryoLife Inc., USA	Human pulmonary and aortic valves	-No mention -No calcification -11% of the explanted valves showed calcification, 0.3% of the total population	[113] [106] [82]
Matrix P® and Matrix P plus N®	(AutoTissue GmbH, Germany)	Porcine tissue sheets	-No mention -No mention	[100] [116]
Arise AV® and Espoir PV®	(Corlife, Germany)	Human pulmonary and aortic valves	-No mention -No calcification	[117] [118]

The first commercially available product in the market was Synergraft^®^ (CryoLife Inc., USA), which showed serious problems in its early models [81]. However, recent models of the product showed very promising results concerning calcification [82]. Matrix P^®^ (AutoTissue GmbH, Germany), a decellularized porcine heart valve used since 2002 in nearly 200 cases. Another product of the same company, Matrix P plus N™ was recently approved [60]. Lately, a clinical trial in 2019 showed promising performance of a newly developed product called ESPOIR® (Corlife, Germany) after a two-year follow-up study [83].

## Conclusions and Future trend

The different species of decellularized valves, decellularization techniques, recellularization or not, storage conditions, characterization tools, duration of follow-up, experimental population and even the confusion in the used terminology, constitute a vast body of complex data, from which it is very difficult to draw solid conclusions. However, still few conclusions may be drawn:

- Different clinical and animal studies showed successful early, mid, and long-term performance of decellularized pulmonary heart valve allografts without calcification for *Ross operation*.
- Different clinical and animals’ studies showed successful early, mid, and long-term results studying the function of decellularized *aortic heart valvesallografts* without calcification.
- Despite the limited number of successful decellularized heart valves xenografts in animal models, only one short and mid-term clinical study with successful results have been reported.
- Although the immunological factor in heart valve calcification is controversial in the literature, in most of the clinical and animal studies it was deemed as an important factor for calcification.
- In the case of calcification of decellularized heart valves, there was no unique pathway different from the calcification mechanisms taking place in native and glutaraldehyde fixed ones.
- The careful selection of the proper heart valve tissue sources (no immunological provoking residues), decellularization technique (no damaged exposed residues of the decellularized tissues, no remnants of dead cells, no remnants of decellularizing agents) and implantation techniques (avoiding suturing during the surgical implantation) could provide a perfect anticalcification potential even with no need for in vitro cell seeding or additional scaffold treatment.
